# Egg preservation in an Eocene stingray (Myliobatiformes, Dasyatidae) from Italy

**DOI:** 10.1080/02724634.2019.1578967

**Published:** 2019-04-09

**Authors:** Federico Fanti, Gabriele Mazzuferi, Giuseppe Marramà

**Affiliations:** 1 Dipartimento di Scienze Biologiche, Geologiche e Ambientali, Università di Bologna, Via Zamboni 67, Bologna 40126, Italy, federico.fanti@unibo.it; 2 Museo Geologico Giovanni Capellini, Alma Mater Studiorum, Università di Bologna, Via Zamboni 63, Bologna 40126, Italy, gabriele.mazzuferi@outlook.it; 3 Department of Palaeontology, University of Vienna, Althanstrasse 14, 1090 Vienna, Austria, giuseppe.marrama@univie.ac.at

Known since the 16th century, Pesciara di Bolca in Italy represents one of the most intensively sampled Eocene marine localities, providing an unparalleled window on the early evolution of modern marine faunas (Friedman and Carnevale, 2018). Although the long-term collecting efforts have resulted in the identification of more than 80 vertebrate families, the complex alpha taxonomy of chondrichthyans, mostly represented by exquisitely preserved individuals, has received attention only in the last few years (Fanti et al., 2016; Marramà et al., 2017, 2018a, 2018b, 2018c, 2018d). Restoration of damage caused by an earthquake brought the historical Bolca collection of the Museo Geologico Giovanni Capellini (Bologna, Italy) under close reexamination. Among others, a complete whiptail stingray of the myliobatiform family Dasyatidae, *Tethytrygon muricatus*, was restored and examined in detail. The use of ultraviolet (UV) light unveiled details of the shape and size of the fins, individual skeletal cartilages, and soft tissues. The individual is interpreted as a sexually mature female based on the absence of claspers and presence of the uterus bearing four eggs. This is the first report of preserved fossilized eggs for stingrays, and in general of eggs in situ in a fossil batoid. Shape, microscopic structure, and relative size of the eggs compared with the overall body size of the specimen indicate an early stage of development of the eggs but also provide a remarkable opportunity to compare fossil and extant representatives of this clade and to further discuss the postulated Eocene ‘nursery’ habitat for the Bolca locality. This fossil demonstrates that a modern reproductive strategy had already been acquired in the early Cenozoic at a body size similar to that of sexually mature extant stingrays.

## MATERIALS AND METHODS

This study is based on a nearly complete and articulated specimen currently housed in the Museo Geologico Giovanni Capellini, Università degli Studi di Bologna (MGGC 7456). The specimen was examined under UV light in order to distinguish the preserved soft tissues from grout or pigments used in historical reconstruction. Measurements were taken to the nearest 0.1  mm, and disc width (DW) is used throughout. Osteological terminology primarily follows Nishida (1990), Lovejoy (1996), and Carvalho et al. (2004). Morphometric terminology is adopted and modified from Last et al. (2016). Criteria for the identification of the preserved elements follow Fanti et al. (2016).

## DESCRIPTION

**FIGURE 1.**
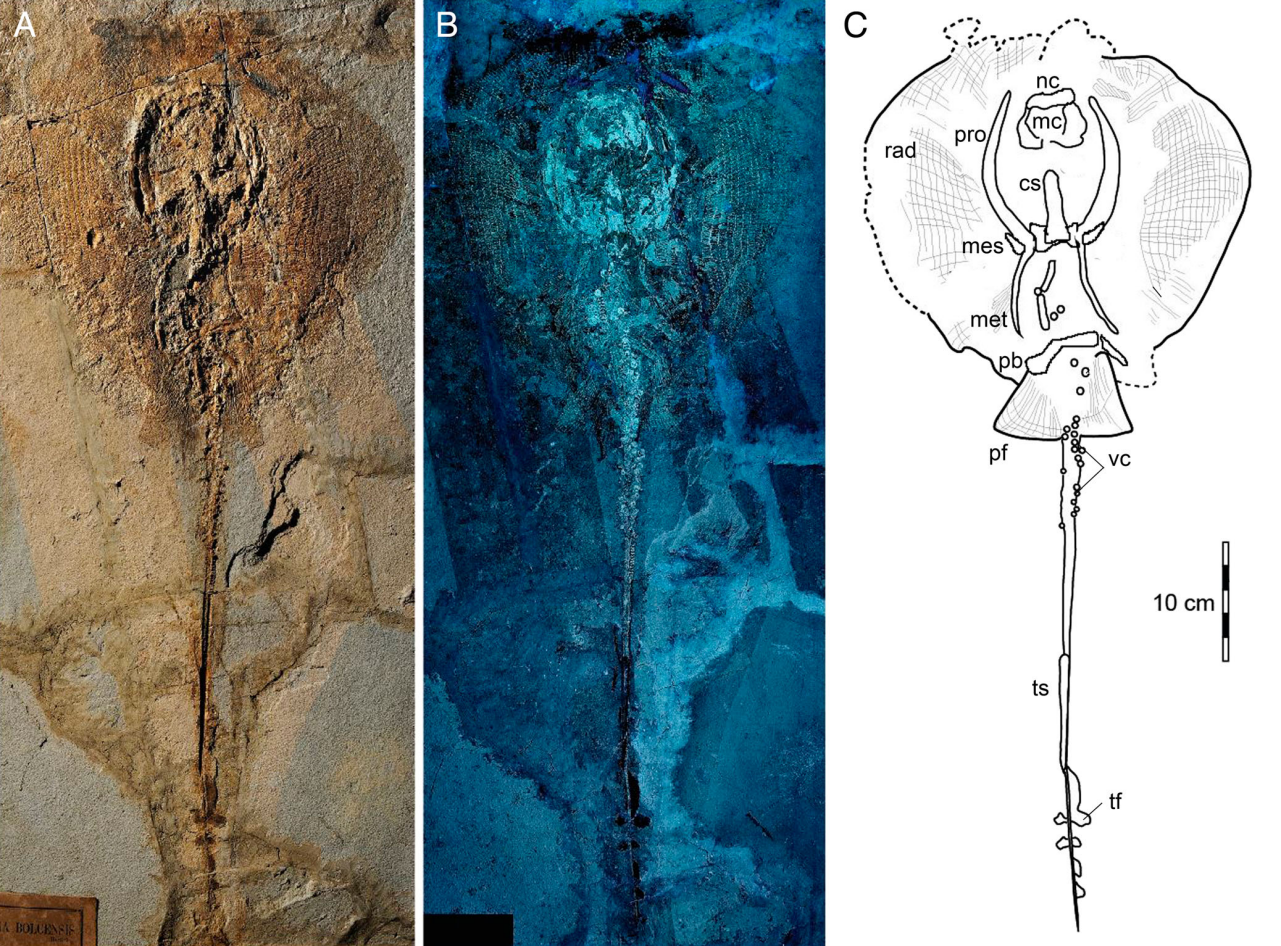
MGGC 7456, *Tethytrygon muricatus* (Volta, 1796) from the Pesciara di Bolca locality. **A**,**B**, photographs of specimen under **A**, natural and **B**, UV light; **C**, interpretive drawing of major anatomical structures preserved. **Abbreviations**: **cs**, cervicothoracic synarcual; **mc**, Meckel’s cartilage; **mes**, mesopterygium; **met**, metapterygium; **nc**, nasal capsules; **pb**, puboischiadic bar; **pf**, pelvic fins; **pro**, propterygium; **rad**, pectoral radials; **tf**, tail folds; **ts**, tail sting; **vc**, vertebral centra.

**FIGURE 2.**
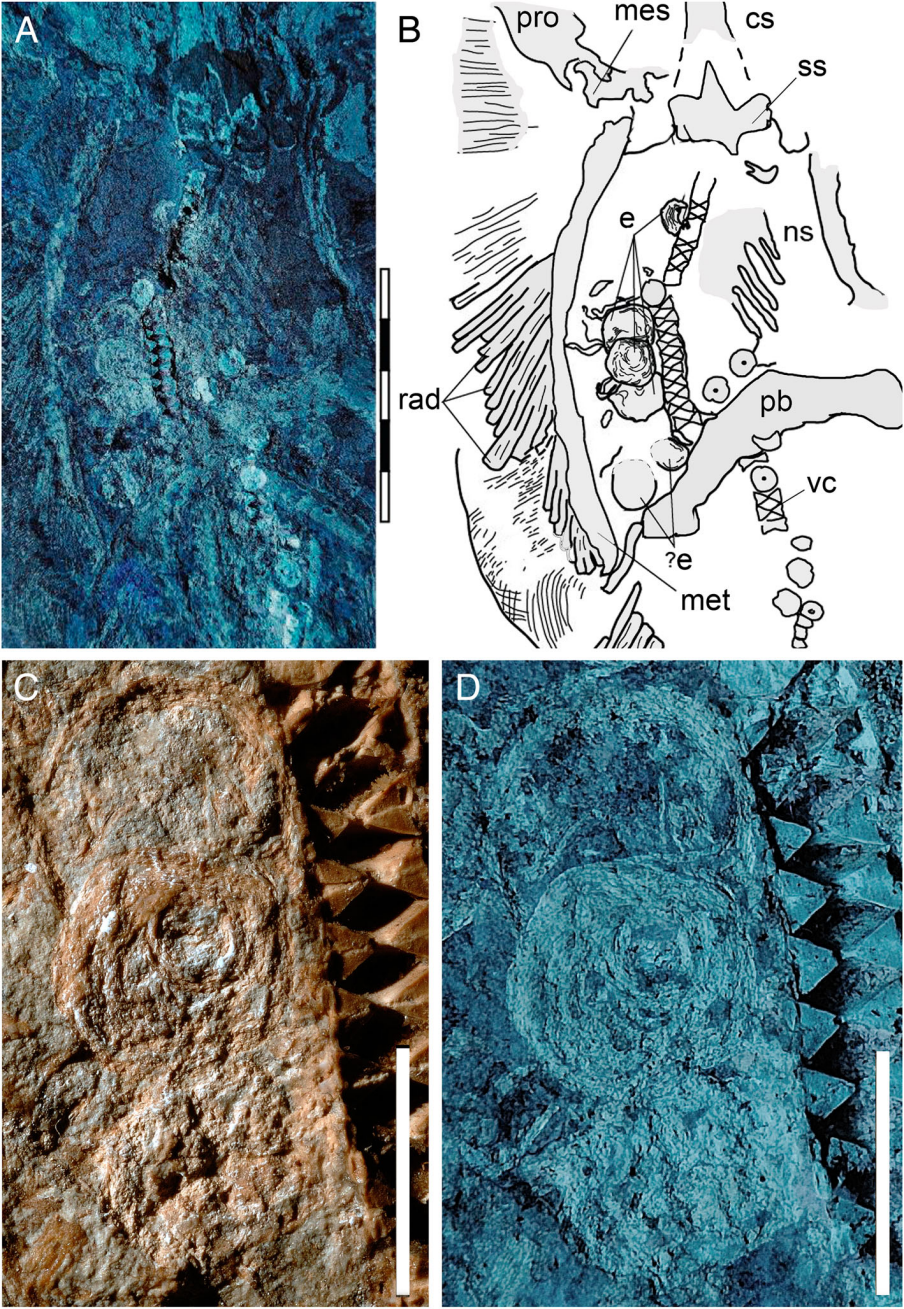
Soft tissue preservation in the abdominal region of MGGC 7456. **A**, UV light photograph showing the duct bearing a minimum of four eggs; **B**, interpretive drawing of anatomical structures; **C**, detail photograph of the three eggs showing exquisite preservation of soft tissues; **D**, the same image under UV light. **Abbreviations**: **cs**, cervicothoracic synarcual; **e**, eggs; **mes**, mesopterygium; **met**, metapterygium; **ns**, neural spines; **pb**, puboischiadic bar; **pro**, propterygium; **rad**, pectoral radials; **ss**, suprascapulae; **vc**, vertebral centra. Scale bars equal 50  mm (**A**,**B**) and 10  mm (**C**,**D**).

The stingray specimen MGGC 7456 (Fig. 1) is preserved in a single slab with the individual exposed predominantly in dorsal view, as suggested by the exposure of the suprascapulae fused to the cervicothoracic synarcual (Fig. 2). The pectoral disc is rhombic in outline and wider than long (disc length about 87% DW) (see Supplemental Data). The chondrocranium and branchial skeleton are incomplete, and elements can be discriminated only under UV light exposure. The specimen lacks the rostral cartilage, as in all stingrays (e.g., Carvalho et al., 2004). Both palatoquadrate and Meckel’s cartilage are incomplete and strongly crushed dorsoventrally onto the otic area. In the pectoral girdle, the propterygia and metapterygia are relatively long, but the latter are slightly smaller in size in comparison with the former. There are about 50 propterygial and 17 mesopterygial radials preserved, but the number of radials on metapterygia is difficult to determine due to taphonomic displacement of these elements. The pelvic girdle is well preserved, as well as the number of pelvic radials per side (ca. 25). No claspers are observed, supporting the identification of the individual as a female. The vertebral column is partially preserved, and about 86 centra are observed (23 of which from scapulocoracoid to pelvic girdle). Individual vertebrae are slightly displaced and appear alternately in their anterior/posterior or lateral views. However, the count of centra in MGGC 7456 is far from being complete because the most complete *Tethytrygon* specimens examined possess 175 to 179 vertebrae in total (see Supplemental Data). The cervicothoracic synarcual is well calcified, with prismatic calcification made of small tesserae (Fig. 2). Its dorsal ridge appears fused to the suprascapulae, suggesting that MGGC 7456 is mostly exposed in dorsal view. Although not well calcified, the thoracolumbar synarcual can be detected posteriorly to the cervicothoracic synarcual, supporting the assignment of MGGC 7456 to stingrays. Long neural spines, postero-obliquely oriented in relation to the centra, are visible in the abdominal cavity just anteriorly and posteriorly to the pelvic girdle. The tail is long, slender, and exceeds half of the total length of the individual or about 180% of DW. The posterior extremity of the tail is stiffened by the presence of a cartilaginous tail rod, which is typically present in dasyatids, potamotrygonids, and pelagic stingrays (Carvalho et al., 2004). As is the case in many stingrays, dorsal fins as well as a completely developed caudal fin are absent. However, in *Tethytrygon*, the caudal fin is reduced to tail folds, which are clearly observable in MGGC 7456, in which UV light has highlighted the presence of dark-pigmented structures along both sides of the tail (Fig. 1). There is a single serrated sting measuring about 90  mm in length, originating at about midlength of the tail, and bearing ca. 45 serrations per side. Ultraviolet photography has improved screening for soft tissue preservation in specimens from Bolca (Fanti et al., 2016; Sansonetti, 2016) and also allowed accurate discrimination and identification of elements. In the area delimited by the left metapterygium and the vertebral column, a longitudinal duct is visible under both natural and UV light (Fig. 2). The duct is ca. 15  mm wide and extends from the posterior margin of the scapulocoracoid to the anal region, where it is partially covered by the puboischiadic bar and the proximal end of the left pelvic fin radials. The position and size of the duct suggest its identification as the uterus (Hamlett et al., 2005; Spieler et al., 2013). No contralateral structure is observed on the right side, which is consistent with the fact that the right ovary is completely absent in most dasyatids (Kardong, 1998; Linzey, 2012). Within the uterus, four subcircular structures ranging from 9 to 17  mm in diameter are preserved between the pectoral and pelvic girdles (Fig. 2). Three of them partly overlap with each other anteriorly, whereas the fourth is located in a more posterior position. Closer examination under both natural and black light reveals minute, concentric laminae ca. 1  mm thick. This feature can be used to distinguish elements preserved within the uterus from isolated vertebrae that are also 40% smaller in diameter.

## TAXONOMY OF MGGC 7456

The taxonomic assignment of MGGC 7456 has been recently discussed by Marramà et al. (2018b), who assigned it to the new genus *Tethytrygon* based on the systematic examination of more than a dozen dasyatid specimens from the Bolca Konservat-Lagerstätte that historically were assigned to ‘*Dasyatis*’ *muricatus* (Volta, 1796) or ‘*Dasyatis*’ *zigni* (Molin, 1861) (‘*D*.’ *zigni* is now considered a junior synonym of ‘*D*.’ *muricatus*; Marramà et al., 2018b). The morphological analysis of MGGC 7456 has revealed diagnostic characters that support its inclusion within the stingray order Myliobatiformes, including the absence of a rostral cartilage, the presence of the thoracolumbar synarcual, and a single, serrated tail sting (e.g., Carvalho et al., 2004; Aschliman et al., 2012). The placement of *T. muricatus* within the Dasyatidae was supported by the ventral terminal cartilage that is free of the axial cartilage and presence of sexual heterodonty, and a combination of several plesiomorphic characters that argues against its placement in other stingray lineages (Marramà et al., 2018b). For example, the presence of tail folds, clearly visible in MGGC 7456 under normal and UV light, excludes its assignment to myliobatids (i.e., pelagic stingrays) and to those dasyatoids characterized by a developed caudal fin (e.g., urolophids and urobatids). An external margin of the mesopterygium that is more or less straight and not fused to radials excludes a closer relationship between *T. muricatus* and *Gymnura* (undulated, not fused to radials) or with the Urolophidae (highly sinuous, fused with radials; e.g., Carvalho et al., 2004). The body proportions and meristic characters of MGGC 7456 fall within the morphological range of *Tethytrygon muricatus* (see Supplemental Data). The morphological and phylogenetic analyses (Marramà et al., 2018b) allows this taxon to be assigned to the subfamily Neotrygoninae.

## POSSIBLE EGGS IN MGGC 7456

Three main reproductive strategies have been reported among extant batoids: oviparity, yolk sac viviparity, and lipid histotrophy (e.g., Musick and Ellis, 2005). Myliobatiforms (including dasyatids) produce lipid-rich histotroph (Hamlett and Koob, 1999; Hamlett et al., 2005; Musick and Ellis, 2005) and supply the developing embryo with proteins and lipid-enriched secretions. Compared with other batoids, this reproductive strategy results in a relatively lower number of large eggs per pregnant female. In dasyatids, the ovaries of mature individuals contain oocytes throughout the year that are embedded in the connective tissue: the onset of ova maturation marks the growth and development of the oocytes. During ovulation, the largest mature ova are released from the ovary, moving to the ostium and oviduct (Maruska et al., 1996; Hamlett and Koob, 1999; Hamlett et al., 2005; Ribeiro et al., 2006; Burns et al., 2014).

We interpret the four oval elements preserved in the body cavity of MGGC 7456 (Fig. 2) as fertilized eggs based on (1) absence of claspers in the parent (indicating that MGGC 7456 is not a male); (2) uniform and relatively large size of the oval elements, consistent with a shared biological origin and incompatible with an alternative interpretation as ingested/food remains; (3) asymmetrical position of the elements, placed on the left side relative to the parasagittal axis of body, consistent with ovary asymmetry (i.e., absence of the right ovary) in the Dasyatidae; and (4) presence of a concentric pattern in the four oval elements, a taphonomic feature reported in fossilized ovarian follicles (e.g., Zheng et al., 2017).

Available data on the fecundity of extant myliobatiform species with viviparous (histotroph) mode of reproduction indicate an average of 4.4 eggs per year, although values range from 1–3 for *Hypanus longus* to 2–10 for *H*. *americanus* (Musick and Ellis, 2005; Ribeiro et al., 2006; variation data within a species and not a population). The relatively large size and low number of eggs in MGGC 7456 (four, possibly six) are consistent with the reproductive physiology observed among extant dasyatids. Similarly, the presence of eggs in this specimen indicates sexual maturity. In relatively large species of living stingrays (i.e., fully grown individuals having a disc width >1  m), sexual maturity in females is observed in individuals commonly longer than 75  cm, as in the case of *Bathytoshia centroura*, *Hypanus guttatus*, and *H*. *americanus* (Capapé, 1993; Henningseng, 2000; Tagliafico et al., 2013). Specimen MGGC 7456 thus documents that a modern reproductive strategy had already been acquired in the early Cenozoic at a similar adult body size to the sexually mature extant stingrays. The living whiptail stingrays occur primarily in shallow coastal waters, lagoons, and estuaries in warm/tropical waters (Last et al., 2016). Living dasyatids utilize coastal and relatively turbid waters as primary nurseries (i.e., habitats where parturition occurs and in which the young live for a short time) (Castro, 1993; Yokota and Lessa, 2006; Heupel et al., 2007; Dale et al., 2011). Although this report does not constitute direct evidence for the Pesciara di Bolca as a nursery for stingrays, because none of the criteria used to recognize a batoid nursery area can be unquestionably detected (Martins et al., 2018; but see also Castro, 1993; Heupel et al., 2007; Pimiento et al., 2010; Fischer et al., 2011; Sallan and Coates, 2014), data presented here might be consistent, at least in part, with this interpretation. A similar conclusion has been drawn for the school shark from Bolca, *Galeorhinus cuvieri*, on the basis of the occurrence of sexually immature juveniles (Fanti et al., 2016), although the presence of juvenile sharks and small shark species in the Bolca paleobiotopes might be related to their competitive advantage in having access to relatively competitor-free trophic niches and food resources, unavailable to larger predators (Marramà et al., 2017).

## Supplementary Material

Supplemental Material
