# Supplementary material for: Egg preservation in an Eocene stingray (Myliobatiformes, Dasyatidae) from Italy
Source: J Vertebr Paleontol. 2019 Apr 9;39(2):e1578967. doi: 10.1080/02724634.2019.1578967 (PMC6817316; doi:10.1080/02724634.2019.1578967)
Supplement: Supplemental Material [file UJVP_A_1578967_SM4250.docx]

Journal of Vertebrate Paleontology

SUPPLEMENTAL DATA

Egg preservation in an Eocene stingray (Myliobatiformes, Dasyatidae) from Italy

FEDERICO FANTI,^1,2^ GABRIELE MAZZUFERI,^2^ and GIUSEPPE MARRAMÀ,^3 1^Dipartimento di Scienze Biologiche, Geologiche e Ambientali, Università di Bologna, Via Zamboni 67, Bologna 40126, Italy, federico.fanti@unibo.it;  ^2^Museo Geologico Giovanni Capellini, Alma Mater Studiorum, Università di Bologna, Via Zamboni 63, Bologna 40126, Italy, gabriele.mazzuferi@outlook.it; ^3^Department of Palaeontology, University of Vienna, Althanstrasse 14, 1090 Vienna, Austria, giuseppe.marrama@univie.ac.at

**
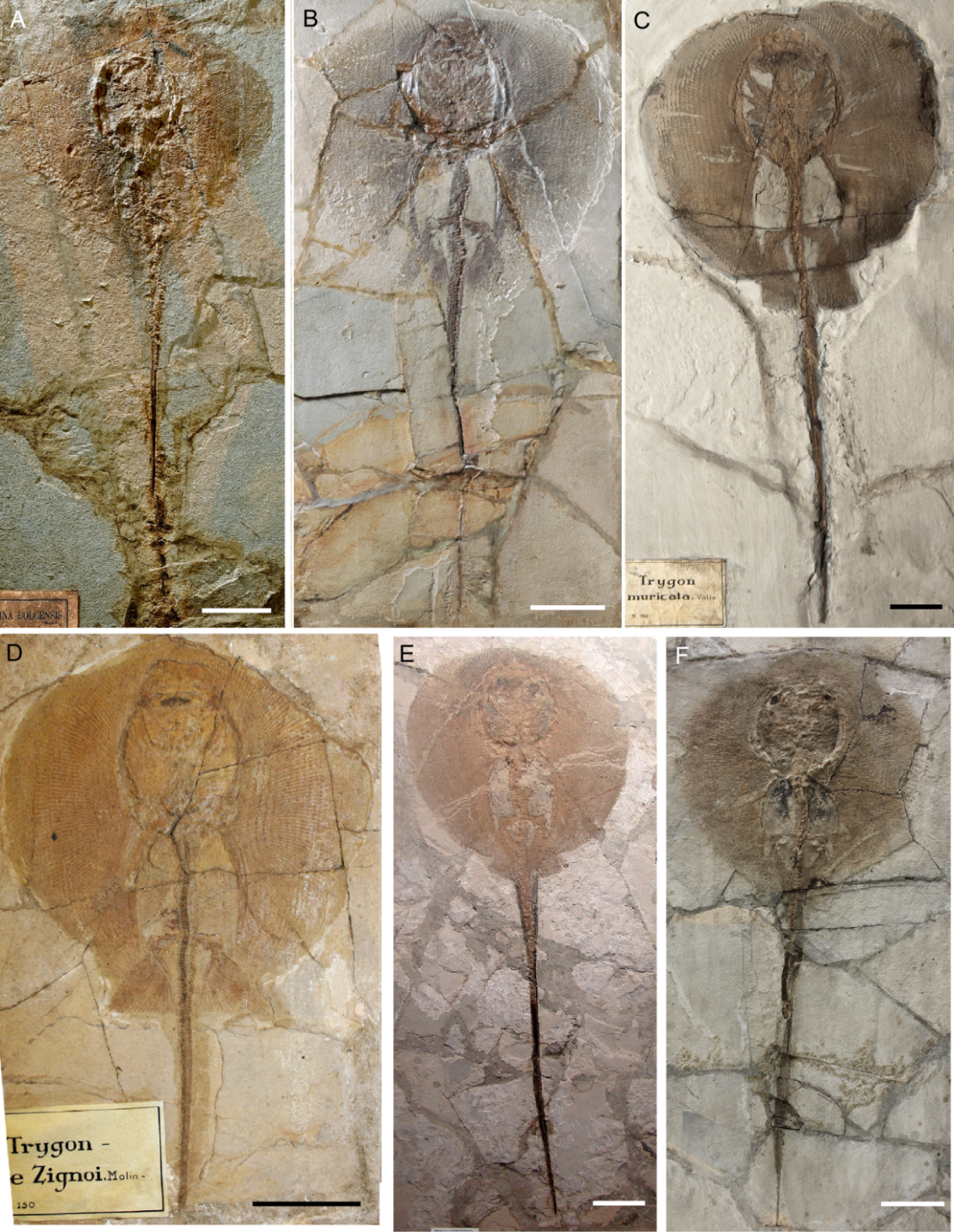
**

FIGURE S1. Comparison between **A**, MGGC 7456, and some of the Bolca stingrays currently assigned to *Tethytrygon muricatus* (Volta, 1796): **B**, MNHN F.Bol564 (holotype); **C**, MGP-PD 160Z; **D**, MGP-PD 150Z; **E**, MCSNV T.1021; **F**, MCSNV II.B.92. **Institutional abbreviations**: **MCSNV**, Museo Civico di Storia Naturale, Verona, Italy; **MGGC**, Museo Geologico Giovanni Capellini, Bologna, Italy; **MGP**-**PD**, Museo di Geologia e Paleontologia dell’Università degli Studi di Padova, Italy; **MNHN**, Museum National d’Histoire Naturelle, Paris, France. Scale bars equal 50 mm.

TABLE S1. Morphometric data for some of the specimens of *Tethytrygon muricatus* (Volta, 1796) from the Eocene Pesciara di Bolca site, examined in Marramà et al. (2018a) also including MGGC 7456. **Institutional abbreviations**: **CMC**, registered private collection of Cerato Massimo Cipriano, Italy; **CMNH**, Carnegie Museum, Pittsburgh, U.S.A.; **MCSNV**, Museo Civico di Storia Naturale, Verona, Italy; **MCZ**, Museum of Comparative Zoology, Harvard University, U.S.A.; **MGGC**, Museo Geologico Giovanni Capellini, University of Bologna, Italy; **MGP**-**PD**, Museo di Geologia e Paleontologia dell’Università degli Studi di Padova, Italy; , U.S.A., Museum National d’Histoire Naturelle, Paris, France.

|  | **MNHN Bol.564 (holotype)** | | **MGP-PD 150Z/151Z** | | **CMNH 4521** | | **CMC2** | | **MCSNV IG.23194** | | **MCSNV IG.186653** | |
| --- | --- | --- | --- | --- | --- | --- | --- | --- | --- | --- | --- | --- |
| **Measurements** | **mm** | **% DW** | **mm** | **% DW** | **mm** | **% DW** | **mm** | **% DW** | **mm** | **% DW** | **mm** | **% DW** |
| Total length | ? | ? | ? | ? | ? | ? | ? | ? | ? | ? | ? | ? |
| Disc length | 243.4 | 86.3 | 128.4 | 89.6 | 193.5 | 94.9 | 128.8 | 88.2 | 574.5 | 95.3 | 293.6 | 104.2 |
| Disc width | 282 | 100.0 | 143.3 | 100.0 | 203.9 | 100.0 | 146 | 100.0 | 603.0 | 100.0 | 281.9 | 100.0 |
| Tail length | ? | ? | ? | ? | ? | ? | ? | ? | ? | ? | ? | ? |
| Preoral length | 37.5 | 13.3 | 22.2 | 15.5 | 26.2 | 12.8 | 22.1 | 15.1 | 89.7 | 14.9 | 47 | 16.7 |
| Mouth-scapulocoracoid distance | 83 | 29.4 | 39.1 | 27.3 | 65.3 | 32.0 | 42.9 | 29.4 | 209.0 | 34.7 | 81.6 | 28.9 |
| Scapulocoracoid width | 51.2 | 18.2 | 30 | 20.9 | 49.1 | 24.1 | 30.7 | 21.0 | 118.8 | 19.7 | 61.3 | 21.7 |
| Pelvic girdle width (width across pelvic-fin base) | 61.3 | 21.7 | 30.5 | 21.3 | 43.1 | 21.1 | 30.6 | 21.0 | ? | ? | 66.9 | 23.7 |
| Sting length | 71.3 | 25.3 | ? | ? | 66.8 | 32.8 | 35.6 | 24.4 | ? | ? | 94.2 | 33.4 |
| Pelvics-tip of tail length | ? | ? | ? | ? | ? | ? | ? | ? | ? | ? | ? | ? |
| Clasper length | ? | ? | ? | ? | ? | ? | ? | ? | 114.8 | 19.0 | ? | ? |
| Neurocranial length | 60.9 | 21.6 | 33.6 | 23.4 | 58.8 | 28.8 | 34.5 | 23.6 | 150.8 | 25.0 | 66.9 | 23.7 |
| Neurocranial width | 41.1 | 14.6 | 21.1 | 14.7 | 40.6 | 19.9 | 24 | 16.4 | ? | ? | 41.4 | 14.7 |
| Pre-sting length | 484.5 | 171.8 | ? | ? | ? | ? | 220.9 | 151.3 | ? | ? | 490.2 | 173.9 |
| Distance from tip of disc to max width disc | 99.9 | 35.4 | 53.3 | 37.2 | 74.6 | 36.6 | 62.9 | 43.1 | 246.2 | 40.8 | 147.3 | 52.3 |
| Prepelvic distance | 199.9 | 70.9 | 109.1 | 76.1 | 157.1 | 77.0 | 102.1 | 69.9 | ? | ? | 218.6 | 77.5 |
| Prescapular distance (head length) | 122.4 | 43.4 | 61.4 | 42.8 | 86.0 | 42.2 | 66.7 | 45.7 | 302.2 | 50.1 | 120.8 | 42.9 |
| Eye diameter | ? | ? | 3.6 | 2.5 | ? | ? | 6.2 | 4.2 | ? | ? | 8.3 | 2.9 |
| Interorbital width | ? | ? | 23 | 16.1 | ? | ? | 12.6 | 8.6 | ? | ? | 49.2 | 17.5 |
| Pelvic fin length | 78.2 | 27.7 | 33.1 | 23.1 | 53.3 | 26.1 | 43.1 | 29.5 | ? | ? | 92.6 | 32.8 |
| Snout to pectoral fin insertion | 225.6 | 80.0 | 112.9 | 78.8 | 170.9 | 83.8 | 110.2 | 75.5 | ? | ? | 241.6 | 85.7 |
| Orbit to pectoral fin insertion | ? | ? | 93 | 64.9 | 128.9 | 63.2 | 82 | 56.2 | ? | ? | 184.5 | 65.4 |
| Snout (preorbital) length | ? | ? | 21.2 | 14.8 | 35.6 | 17.5 | 26.8 | 18.4 | ? | ? | 52.4 | 18.6 |
| Pectoral-fin insertion to sting | 247.9 | 87.9 | ? | ? | ? | ? | 103.2 | 70.7 | ? | ? | 249.7 | 88.6 |
| Propterygial radials | 50 | ? | 51 | ? | ? | ? | 51 | ? | 51 | ? | 53 | ? |
| Mesopterygial radials | ? | ? | 19 | ? | ? | ? | 17 | ? | 16 | ? | 19 | ? |
| Metapterygial radials | ? | ? | 43 | ? | ? | ? | 40 | ? | 42 | ? | 45 | ? |
| Total pectoral radials | ? | ? | 113 | ? | ? | ? | 108 | ? | 109 | ? | 117 | ? |
| Pelvic radials | 25 | ? | 27 | ? | 26 | ? | 25 | ? | 25 | ? | 25 | ? |
| Vertebrae from scapulocoracoid to pelvic girdle | 25 | ? | 24 | ? | 25 | ? | 23 | ? | ? | ? | 24 | ? |
| Vertebrae from pelvic girdle to sting | ? | ? | ? | ? | ? | ? | 102 | ? | ? | ? | 109 | ? |
| Vertabrae posterior to sting | ? | ? | ? | ? | ? | ? | 54 | ? | ? | ? | 46 | ? |
| Total vertebrae | ? | ? | ? | ? | ? | ? | 179 | ? | ? | ? | 179 | ? |
| Sting serrations per side | ? | ? | ? | ? | ? | ? | 24 | ? | ? | ? | 30 | ? |

|  | **MCSNV T.1020/1** | | **MCSNV VII.B.92/3** | | **MCZ 13183** | | **MGGC 7456 (studied specimen)** | | **MGP-PD 159/160** | |
| --- | --- | --- | --- | --- | --- | --- | --- | --- | --- | --- |
| **Measurements** | **mm** | **% DW** | **mm** | **% DW** | **mm** | **% DW** | **mm** | **% DW** | **mm** | **% DW** |
| Total length | 759.2 | 263.0 | 627.2 | 249.5 | ? | ? | **728.7** | **252.4** | ? | ? |
| Disc length | 279.9 | 97.0 | 236.8 | 94.2 | 110.1 | 90.2 | **250.7** | **86.8** | 252.2 | 89.1 |
| Disc width | 288.7 | 100.0 | 251.4 | 100.0 | 122.1 | 100.0 | **288.7** | **100.0** | 283 | 100.0 |
| Tail length | 533.3 | 184.7 | 428.3 | 170.4 | ? | ? | **518.2** | **179.5** | ? | ? |
| Preoral length | 51.4 | 17.8 | ? | ? | 18.2 | 14.9 | **?** | **?** | 38 | 13.4 |
| Mouth-scapulocoracoid distance | 78 | 27.0 | ? | ? | 37.7 | 30.9 | **?** | **?** | 87.3 | 30.8 |
| Scapulocoracoid width | 59 | 20.4 | 57.4 | 22.8 | 24.2 | 19.8 | **54.9** | **19.0** | 68.6 | 24.2 |
| Pelvic girdle width (width across pelvic-fin base) | 63.6 | 22.0 | 56.6 | 22.5 | 34.6 | 28.3 | **64.3** | **22.3** | 66.1 | 23.4 |
| Sting length | 82.5 | 28.6 | ? | ? | ? | ? | **89.6** | **31.0** | 91.8 | 32.4 |
| Pelvics-tip of tail length | 452.5 | 156.7 | 356.4 | 141.8 | ? | ? | **428.5** | **148.4** | ? | ? |
| Clasper length | ? | ? | ? | ? | ? | ? | **?** | **?** | ? | ? |
| Neurocranial length | 71.5 | 24.8 | 57.3 | 22.8 | ? | ? | **60** | **20.8** | 69.9 | 24.7 |
| Neurocranial width | ? | ? | 46.1 | 18.3 | ? | ? | **?** | **?** | 42.1 | 14.9 |
| Pre-sting length | 472.5 | 163.7 | 361 | 143.6 | 213 | 174.4 | **469.2** | **162.5** | 443.1 | 156.6 |
| Distance from tip of disc to max width disc | 110.6 | 38.3 | 112 | 44.6 | ? | ? | **113.5** | **39.3** | 95 | 33.6 |
| Prepelvic distance | 224.4 | 77.7 | 199.9 | 79.5 | 97.3 | 79.7 | **211.6** | **73.3** | 203.6 | 71.9 |
| Prescapular distance (head length) | 126.8 | 43.9 | 132.2 | 52.6 | ? | ? | **144.1** | **49.9** | 126 | 44.5 |
| Eye diameter | 13.5 | 4.7 | 10.5 | 4.2 | ? | ? | **?** | **?** | ? | ? |
| Interorbital width | 36.7 | 12.7 | 45.1 | 17.9 | ? | ? | **?** | **?** | ? | ? |
| Pelvic fin length | 85.6 | 29.7 | 76.2 | 30.3 | ? | ? | **70.5** | **24.4** | 69.6 | 24.6 |
| Snout to pectoral fin insertion | 248 | 85.9 | 221.5 | 88.1 | ? | ? | **215.1** | **74.5** | 239.2 | 84.5 |
| Orbit to pectoral fin insertion | 197.1 | 68.3 | 175.9 | 70.0 | ? | ? | **?** | **?** | ? | ? |
| Snout (preorbital) length | 43.9 | 15.2 | 42 | 16.7 | ? | ? | **?** | **?** | ? | ? |
| Pectoral-fin insertion to sting | 220.8 | 76.5 | ? | ? | ? | ? | **216.6** | **75.0** | 214.3 | 75.7 |
| Propterygial radials | 50 | ? | 49 | ? | ? | ? | **50** | **?** | 50 | ? |
| Mesopterygial radials | 20 | ? | 16 | ? | ? | ? | **17** | **?** | 18 | ? |
| Metapterygial radials | 44 | ? | 45 | ? | ? | ? | **?** | **?** | 45 | ? |
| Total pectoral radials | 114 | ? | 110 | ? | ? | ? | **?** | **?** | 113 | ? |
| Pelvic radials | 25 | ? | 24 | ? | ? | ? | **25** | **?** | 25 | ? |
| Vertebrae from scapulocoracoid to pelvic girdle | 26 | ? | 25 | ? | ? | ? | **23** | **?** | 24 | ? |
| Vertebrae from pelvic girdle to sting | 105 | ? | ? | ? | ? | ? | **?** | **?** | 100 | ? |
| Vertabrae posterior to sting | 45 | ? | ? | ? | ? | ? | **?** | **?** | ? | ? |
| Total vertebrae | 176 | ? | 175 | ? | ? | ? | **?** | **?** | ? | ? |
| Sting serrations per side | 33 | ? | 43 | ? | ? | ? | **45** | **?** | 39 | ? |
